# Gaps in transition readiness measurement: a comparison of instruments to a conceptual model

**DOI:** 10.1515/jtm-2022-0002

**Published:** 2022-07-12

**Authors:** Katherine South, Maureen George, Arlene Smaldone

**Affiliations:** Columbia University School of Nursing, New York, NY, USA

**Keywords:** adolescent and young adult (AYA), conceptual framework, healthcare transition, transition readiness measurement

## Abstract

**Objectives:**

Measuring transition readiness is important when preparing young people with chronic illness for successful transition to adult care. The Expanded Socioecological Model of Adolescent and Young Adult Readiness to Transition (Expanded SMART) offers a holistic view of factors that influence transition readiness and outcomes. The aim of this study was to examine conceptual congruency of transition readiness instruments with the Expanded SMART to determine the breadth and frequency of constructs measured.

**Methods:**

PubMed was searched to identify observational and experimental studies that measured transition readiness across chronic illnesses. Selected instruments were first evaluated on their development and psychometric properties. Next, reviewers independently mapped each instrument item to Expanded SMART constructs: knowledge, skills/self-efficacy, relationships/communication, psychosocial/emotions, developmental maturity, beliefs/expectations, goals/motivation. If items did not map to a construct, a new construct was named inductively through group discussion.

**Results:**

Three instruments (TRAQ [20 items], STARx [18 items] and TRxANSITION Index [32 items]), reported in 74 studies, were identified. Across instruments, most items mapped to three constructs: skills/self-efficacy, developmental maturity, and knowledge. The psychosocial constructs of goals/motivation and psychosocial/emotions were underrepresented in the instruments. No instrument mapped to every model construct. Two new constructs: *independent living* and *organization* were identified.

**Conclusions:**

Constructs representing transition readiness in three frequently used transition readiness instruments vary considerably from Expanded SMART, a holistic conceptual model of transition readiness, suggesting that conceptualization and operationalization of transition readiness is not standardized. No instrument reflected all conceptual constructs of transition readiness and psychosocial constructs were underrepresented, suggesting that current instruments may provide an incomplete measurement of transition readiness.

## Introduction

Today, 90% of youth diagnosed with a chronic illness in childhood will reach adulthood [1] with an estimated one million of these adolescent and young adult (AYA) patients in the United States establishing care in the adult-oriented healthcare setting each year [2, 3]. As this population ages, pediatric and adult care providers must work together to prepare emerging young adults to manage their healthcare needs independently in the adult care setting. Healthcare transition, the planned and purposeful movement of AYAs with chronic illness from pediatric to adult care, is key to supporting the lifelong health and wellbeing of this population [4].

To successfully transition to adult care, AYAs with chronic illness must be ready to assume primary responsibility for managing treatments and coordinating their healthcare [5]. When AYAs are not prepared to navigate the adult care setting and manage their care with less involvement from their parents or caregivers, gaps in continuity of recommended care [6], [7], [8] and clinical deterioration [9], [10], [11], [12], [13], [14], [15] can occur. To avoid these negative consequences, it is recommended that transition planning and transfer to adult care be tailored to an individual AYA’s readiness, rather than based on age alone [16, 17].

Determining an AYA’s healthcare transition readiness is an integral part of ensuring a smooth and successful transition to adult care. Transition readiness, the likelihood that an AYA will be able to successfully complete their transition to adult care [18], is often measured with transition readiness instruments [19]. Knowing an individual’s level of transition readiness is key to determining when an AYA is ready to initiate their transfer to adult care. Measures of transition readiness can also be used to evaluate the effectiveness of transition preparation interventions [20], track progress over time and identify barriers to a smooth transition [21]. While few studies have examined outcomes by transition readiness, in a study of AYA renal transplant recipients, increased transition readiness was associated with improved medication adherence and lower emergency room utilization [22].

Healthcare transition readiness is a complex concept which is influenced by many factors [23]. The Expanded Socioecological Model of AYA Readiness to Transition (SMART) model [24] identifies seven modifiable factors that have been hypothesized to influence transition readiness and outcomes: knowledge, skills/self-efficacy, relationships/communication, psychosocial/emotions, developmental maturity, beliefs/expectations and goals/motivation [24]. Measurement of these constructs is especially important as they are most likely to change over time and be influenced by transition preparation interventions [23].

One of the primary challenges in measuring transition readiness is a lack of consensus on the most important factors which contribute to this construct [3, 22, 25]. No research to our knowledge has determined the extent to which items of transition readiness instruments align with a conceptual model and the breadth (number of constructs represented in an instrument) and frequency (number of items which align with a given construct) of constructs covered by transition readiness instruments. Thus, the aim of this study was to map items of three commonly used transition readiness instruments to the Expanded SMART to determine the breadth and frequency of model constructs represented by each instrument.

## Materials and methods

### Instrument selection

A literature search in PubMed using the search term “transition readiness” was used to identify studies from 2007 to 2021 which used an instrument to measure transition readiness. Inclusion criteria stipulated that only the youth version of an instrument would be evaluated if an accompanying caregiver version was also available. Studies which used a condition specific transition readiness instrument were excluded as a generalized approach to transition preparation has been considered a more equitable and cost effective option [26]. Of 279 studies retrieved, one researcher (KS) identified those experimental and observational studies that measured transition readiness. Frequency of citation of various instruments was used to generate a list of instruments which are most often used in transition research. Data extracted from these studies included the chronic condition(s) of the participants and the transition readiness instrument used. Using these data, three instruments were selected based on their utility across multiple chronic illnesses.

### Evaluation

Following the selection process, each instrument and its supporting publications were reviewed. Information on the process of each instrument’s development was extracted from the supporting publications and tracked using an Excel spreadsheet. Reported psychometric properties (Cronbach’s alpha or interrater reliability) for each selected instrument were extracted and evaluated by the research team and the chronic medical condition(s) for which instruments were used was catalogued. Following instrument selection and evaluation of psychometric properties, each author independently mapped items from each instrument to one of the seven Expanded SMART constructs. If an item did not map to an *a priori* construct, a newly identified construct was named. Consensus was reached with group discussion.

## Results

Six instruments were identified by the literature review: the Transition Readiness Assessment Questionnaire (TRAQ) [20], the Readiness for Transition Questionnaire (RTQ) [27], the Self-management and Transition to Adulthood with Rx=treatment (STARx) [25], the Transition Q [28], the Am I on TRAC [29], and the TRxANSITION Index [30]. The TRAQ was cited in most studies (n=59) followed by the STARx (n=12), the RTQ (n=5) and the TRxANSITION Index (n=3). The TRAQ, STARx and TRxANSITION Index exhibited the most utility across different chronic conditions. Of 21 diagnostic categories representing chronic illnesses where transition readiness was measured, the TRAQ represented 18 diagnostic categories, followed by 17 for the STARx, and 11 for the TRxANSITION Index. Diagnostic categories represented by each instrument are presented in Table 1.

**Table 1:** Diagnostic categories representing chronic illnesses in which transition readiness was measured.

Diagnostic category Number (n) of studies identified using the instrument	TRAQ (n=59)	STARx (n=12)	TRxANSITION Index (n=3)	RTQ (n=5)	Transition Q (n=2)	Am I on TRAC (n=1)
Allergy and immunology	X	X				
Cardiovascular	X	X	X	X	X	
Craniofacial abnormalities		X				
Dermatology		X				
Endocrine	X	X	X			X
Gastrointestinal	X	X	X	X		
Genetic disorders	X	X	X			
Genitourinary	X					
Gynecology	X					
Hematology	X	X	X	X		
Infectious disease	X	X	X			
Musculoskeletal		X				
Neurodevelopmental	X					
Neurologic	X	X				
Neuromuscular	X	X				
Oncology	X	X	X			
Physical disability	X	X				
Pulmonary	X	X	X			
Renal	X	X	X			
Rheumatology	X	X	X		X	
Transplant	X		X	X		

Based on the number of chronic illnesses in which the instrument(s) had been used, the TRAQ, the STARx and the TRxANSITION Index were selected for further evaluation. During their development, all three instruments had been tested on populations of AYAs with various chronic illnesses; in some cases, researchers applied testing of the transition readiness instrument to samples representing two or more chronic conditions such as: sickle cell disease, diabetes [25, 30, 31], inflammatory bowel disease, chronic kidney disease, systemic lupus erythematous, hypertension [25, 30], cerebral palsy, spina bifida, cystic fibrosis [25, 31], renal transplant [30], heart disease, lung disease, physical disabilities, burns, skin disease, cancer, neurologic disorders, genetic conditions, craniofacial abnormalities, human immunodeficiency virus/acquired immunodeficiency syndrome (HIV/AIDS) [25], seizure disorders, autism, and developmental disabilities [31].

The TRAQ is a 20 item self-report measure with a reported Cronbach’s alpha of 0.94 [20]. Subscales on the TRAQ include: appointment keeping, tracking health issues, managing medications, talking with providers and managing daily activities [20]. The five response options on the TRAQ are based on the transtheoretical stages of change model [32] and range from “I do not know how to do this” (lowest readiness) to “I always do this when I need to” (highest readiness) [31].

The STARx is an 18 item self-report measure with a reported Cronbach’s alpha of 0.8 [25]. The STARx contains six subscales: medication management, provider communication, engagement during appointments, disease knowledge, adult health responsibilities and resource utilization [25]. Wording of the response options varies based on the question and the item scores range from zero (lowest readiness) to four (highest readiness) [33]. An additional response option of “I do not take medications” is available for medication-related questions [33].

The TRxANSITION Index contains 32 items and is delivered by a clinician in an interview format with a reported interrater reliability of 0.71 (no reported Cronbach’s alpha) and 10 subscales: type of illness, medications, nutrition, adherence, self-management activities, informed reproduction, trade/school, insurance, ongoing support and new health providers [30]. Each of the 10 subscales can be scored as a maximum of one point with total overall score ranging from zero (lowest readiness) to 10 (highest readiness) [30].

During the development processes of the TRAQ, STARx and TRxANSITION Index, target audience feedback was obtained through focus groups or interviews with AYAs with chronic conditions [25, 30, 31].

**Figure 1:**
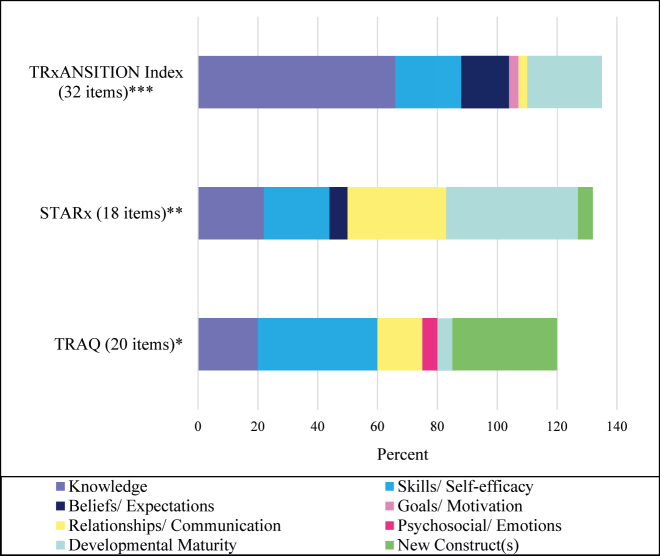
Proportion of items on the TRxANSITION Index, STARx and TRAQ mapping to expanded SMART model constructs. Scale range extends beyond 100% to accommodate items from each instrument which mapped to more than one construct ***9 items mapped to >1 construct; **5 items mapped to >1 construct; *4 items mapped to >1 construct.

Figure 1 illustrates the proportion of items which mapped to each model construct by instrument. No instrument represented all model constructs. The TRxANSITION Index items mapped to six Expanded SMART model constructs, while the TRAQ and STARx items mapped to five constructs. In each instrument some items mapped to more than one construct. For example, the item “Do you usually call/email your MD if you have a question or need to speak to them?” on the TRxANSITION Index mapped to both relationships/communication and developmental maturity.

Across instruments, items most frequently mapped to four constructs: knowledge, skills/self-efficacy, relationships/communication, and developmental maturity. At least one item from each instrument mapped to each of these constructs. Items relating to knowledge asked an AYA for specific facts relating to their condition or treatments, while skills/self-efficacy items asked about actions taken related to taking care of one’s health, such as calling to schedule medical appointments. Items mapping to relationships/communication related to an AYA’s communication with their healthcare team and developmental maturity items focused on an AYA’s level of confidence or ability related to independently taking on responsibility for their care.

Fewer items mapped to psychosocial domains such as beliefs/expectations and goals/motivation. Beliefs/expectations items focused on what an AYA perceived as the consequences for various health behaviors, such as not taking medications as prescribed. Five items on the TRxANSITION Index and a single item on the STARx mapped to beliefs/expectations. Goals/motivation items focused on an AYA’s plans for future work or education while psychosocial/emotions focused on emotional needs or concerns. The constructs goals/motivation and psychosocial/emotions were least frequently represented; each only mapped to one item on one instrument.

Distribution of items across the Expanded SMART model constructs varied across items with certain constructs represented more frequently than others (Figure 1). For example, knowledge was most frequently represented on the TRxANSITION Index, with 21 of 32 items (66%) mapping to this construct. On the TRAQ, 8 of 20 (40%) items mapped to skills/self-efficacy. For the STARx, 8 of 18 (44%) items mapped to developmental maturity.

Across instruments, eight items (seven TRAQ items; one STARx item) did not map to an expanded SMART model construct. Two new constructs, *independent living* and *organization*, were inductively identified. Items mapping to independent living focused on an AYA’s ability to perform day to day tasks, often not directly related to illness self-management, such as cleaning one’s room or budgeting for household expenses. Items mapping to organization assessed an AYA’s use of organizational strategies to manage their health needs such as using a pill box or writing down a list of questions for their provider prior to an appointment. Group discussion was used to name these new constructs and ensure they contained characteristics which differentiated them from existing constructs. While both new constructs were similar to the model construct of developmental maturity, we determined that the new constructs were more specific and described particular tasks or skills to a greater extent compared to the more general construct of developmental maturity. In particular, the new construct of independent living captured constructs not directly related to illness self-management.

## Discussion

Our study adds to the current literature examining transition readiness instruments by mapping items of three widely used transition readiness instruments to the Expanded SMART conceptual model. No instrument captured all Expanded SMART model constructs. Acquisition of knowledge and skill were represented by all measures and psychosocial constructs (i.e.: goals/motivation, psychosocial/emotions) were least represented. This is cause for concern; while disease knowledge and self-management skills are necessary, they are not sufficient to ensure successful healthcare transition if the AYA is not motivated, lacks developmental maturity or family and provider support [34, 35]. This supports Schwartz and colleagues’ contention that transition readiness instruments are not one size fits all [36].

The variation in breadth and frequency of Expanded SMART constructs represented by the TRAQ, STARx and TRxANSITION Index highlights a larger problem in transition readiness measurement: lack of a clear conceptual definition of transition readiness [3, 37, 38]. Some instruments place emphasis on an AYA’s behaviors while others aim to measure knowledge and skills [3]. Additionally, our new construct independent living contains more general items which do not directly ask about illness self-management behaviors; only items on the TRAQ mapped to independent living. These differences in operationalization and conceptualization of transition readiness make it difficult, if not impossible, to compare subscales across different instruments, thus limiting generalizations across the body of healthcare transition research [3, 38].

This inconsistent conceptualization of transition readiness is common in the healthcare transition literature. A systematic review of 10 transition readiness instruments, which did not compare the instruments to a conceptual model, demonstrated similar inconsistencies in construct conceptualization, with the majority of included instruments measuring knowledge and skills/self-management [36, 38] but poorly representing the psychosocial components of transition readiness. An inconsistent conceptual definition of transition readiness is also supported by the findings of a recent systematic review of factors influencing transition readiness [39]. The 33 included studies identified a wide range of factors associated with transition readiness ranging from psychosocial factors such as motivation, locus of control and social support to disease knowledge, self-efficacy, disease responsibility and parent involvement in care [39]. This finding highlights a gap in the available instruments and supports the development of more comprehensive, theory guided instruments. Development of conceptually based instruments which comprehensively measure transition readiness is needed.

This work is not without limitations. Because we stipulated that included instruments be generic and applicable to multiple diseases, it is possible that disease specific instruments more consistently measure transition readiness and capture valuable information that non-disease specific instruments miss [39, 40]. Additionally, the broad search strategy used as well as only reviewing observational and experimental studies may have missed some instruments for inclusion. Finally, while some items on the questionnaires did not relate directly to healthcare transition readiness or illness self-management (i.e.: “Do you help keep home/room clean or clean up after meals?” on the TRAQ [20]), we mapped all items to a construct as these items are included on the original questionnaires.

Mapping items from the TRAQ, STARx and TRxANSITION Index to the Expanded SMART demonstrated that these instruments showed moderate to comprehensive breadth of model constructs covered but the frequency with which items aligned to different model constructs varied, with psychosocial constructs generally receiving least emphasis. As a result, clinicians must be intentional in their selection of transition readiness instruments [36]. Clinicians should be aware of which constructs their selected instrument predominately measures and tailor instrument choice based on patient needs and goal of the assessment. Additionally, this work demonstrates a lack of standardization in how transition readiness is defined and operationalized and the absence of theory guiding transition readiness instrument development and research. Future research should examine how disease specific transition readiness instruments align with conceptual models and consider developing new or redesigning current instruments to be more comprehensive.
